# Transdiagnostic Clinical Global Impression Scoring for Routine Clinical Settings

**DOI:** 10.3390/bs7030040

**Published:** 2017-06-27

**Authors:** Boadie W. Dunlop, Jaclyn Gray, Mark H. Rapaport

**Affiliations:** Department of Psychiatry and Behavioral Sciences, Emory University School of Medicine, 12 Executive Park Drive, 3rd Floor, Atlanta, GA 30329, USA; jaclyn.gray@emory.edu (J.G.); mark.h.rapaport@emory.edu (M.H.R.)

**Keywords:** mood disorders, psychotic disorders, anxiety disorders, pharmacotherapy, psychotherapy, rating scale

## Abstract

Although there is great interest in the improving the ability to track patients’ change over time in routine clinical care settings, no standardized transdiagnostic measure is currently available for busy clinicians to apply. The Clinical Global Impression (CGI) scales are simple measures widely used as outcomes in psychiatric clinical trials. However, the CGI suffers from poorly defined scoring anchors. Efforts to improve the anchors by enhancing the anchor descriptions have proven useful but are limited by being disease-specific, thereby acting as a barrier to the routine clinical adoption of the CGI. To inform the development of more broadly applicable CGI scoring anchors, we surveyed 24 clinical trial investigators, asking them to rank-order seven elements that inform their CGI-Severity (CGI-S) scoring. Symptom severity emerged as the most important element in determining CGI-S scores; the functional status of the patient emerged as a second element. Less importance was given to self-report symptom scores, staff observations, or side effects. Relative rankings of the elements’ importance did not differ by investigators’ experience nor time usually spent with patients. We integrated these results with published illness-specific CGI anchors to develop the Transdiagnostic CGI (T-CGI), which employs standardized scoring anchors applicable across psychiatric illnesses. Pending validity and reliability evaluations, the T-CGI may prove well-suited for inclusion in routine clinical settings and for incorporation into electronic medical records as a simple and useful measure of treatment efficacy.

## 1. Introduction

Fundamental to the treatment of psychiatric disorders is the ability of clinicians to determine whether an intervention is helping a patient recover from their illness. In clinical trials, accurate and consistent measurement of improvement is crucial for determining the potential efficacy of a new treatment. However, routine clinical settings have lagged in adopting standardized measures of change, despite the demonstrated value of measurement-based care for improving patient outcomes [1,2,3]. Less than 20% of psychiatrists routinely use symptom rating scales as part of their clinical practice [4]. This low rate of adoption stems from the time-consuming and disease-specific rating scales that are not practical options in busy clinical settings, as well as clinicians’ perceptions that existing rating scales have limited clinical utility [4,5]. The development of a transdiagnostic measure that easily and reliably captures illness severity and change over time would address this unmet clinical need.

Historically, symptom rating scales have been the primary tools used to assess efficacy, because they have well-established psychometric properties and evaluate a range of symptoms. However, several concerns about the reliability and validity of the various measures commonly employed in clinical trials exist, and rating scale scores may be confounded by side effects of medications (such as changes in appetite or sleep) that are scored as new symptoms by masked raters or by patients on self-report questionnaires [6,7]. Moreover, meaningful assessment of treatment efficacy requires consideration of factors beyond symptom change alone, such as quality of life and level of functioning [8,9]. How patients perceive their own quality of life is only partially explained by symptom rating scale scores [10], and the scales fail to fully capture changes in functional status [11]. Although functioning typically improves with symptom reduction, these concepts are not always concordant, and functional changes [12] and quality of life gains [13] often lag behind symptom change.

The Clinical Global Impression (CGI) scales were developed as simplified global measures to reflect the clinician’s overall impression of a patient’s condition (CGI-Severity, CGI-S, rated 1–7 from “normal” to “among the most extremely ill” and change over time (CGI-Improvement, CGI-I, rated 1–7 from “very much improved” to “very much worse” [14]. The appeal of the CGI measures is their easy translation to clinical care; they represent a common heuristic used by clinicians in evaluating patients and making treatment decisions [15]. However, an important limitation of the original CGI scales is their lack of well-defined anchor points [15]. Revisions of classic symptom rating scales have identified ambiguous or absent anchor point descriptions as a significant source of unreliability in scoring, addressed by adding explicit descriptions for the numerical scores [16,17,18]. For the CGI, the rater’s experience may also be a source of variability, given the scale’s instruction to “consider his [sic] total clinical experience with the given population” in making the rating [14]. More detailed anchor point descriptions for the CGI scales are therefore necessary to improve inter-rater reliability, and have been developed by several groups for illness-specific versions of the CGI [19,20,21,22,23,24,25,26].

In addition to concerns about inter-rater reliability, psychometric evaluations of the CGI have identified potential problems with validity, scaling, and test-retest reliability, in some disease populations [27,28,29,30]. Despite these concerns, Leon and colleagues found the CGI measures to have good internal consistency and concurrent validity, which could be further improved by more rigorous rater training and more well-structured anchor points [31]. Indeed, the CGI scales have been used as the primary outcome measure in clinical trials for a variety of conditions, including major depression [32], social phobia [33], post-traumatic stress disorder [34], panic disorder [35], binge-eating disorder [36], and complicated grief [25,26]. A recent modification of the CGI that aimed to improve scoring reliability in clinical trials, the Structured Interview Guide for Global Impressions [37], requires approximately ten minutes to complete. Although ten minutes to administer a scale during a clinical trial visit is not excessive, this would amount to 33–50% of the time allotted for outpatient psychiatric appointments, making it impractical for routine clinical settings.

In summary, the CGI scales appear to have potential utility as a rating tool for routine clinical settings, but adoption would be improved by having anchors that could be applied across illnesses, and reliability could be increased by clarifying the anchor points used for scoring. In pursuit of these goals, we examined the importance of elements used in making CGI-S assessments by conducting a survey of investigators who conduct CGI ratings during clinical trials for major depression. We aimed to develop anchors for scoring the CGI across psychiatric illnesses (transdiagnostically) by integrating the results of this survey with published scoring guidelines for the CGI used in trials of several psychiatric disorders.

## 2. Materials and Methods

In order to inform the creation of scoring anchors for a transdiagnostic CGI, we engaged a group of clinical trialists with expertise across mood, anxiety and psychotic disorders at an investigator’s meeting for a study of major depressive disorder. We created a questionnaire to assess the elements they considered to be most important when formulating a CGI-S score. The questionnaire was approved by the Emory Institutional Review Board and distributed during the meeting.

The questionnaire was comprised of a single page that instructed investigators to rank order from 1 to 7 the importance of seven elements they use when determining a CGI-S rating. The questionnaire stated that “1” represented the most important element and “7” the least important. The questionnaire instructed the investigators to answer as if they were evaluating a patient at week 4 of an 8-week placebo-controlled trial of a medication. Because the meeting was for a depression trial we chose the following seven elements, presented as shown in Table 1. The questionnaire included a blank space asking investigators to write any additional elements relevant to their CGI-S ratings other the seven listed.

In addition to the elements contributing to CGI ratings, the questionnaire had specific printed questions, asking (1) their academic degree; (2) the number of studies for which the investigator had performed CGI ratings; and (3) how much time they typically spent with a patient at a mid-trial visit (with categories of <10 min, 10–19 min, 20–29 min, and ≥30 min). No personally identifying information of the investigators was collected.

Questionnaire data were analyzed in SPSS version 24.0 (SPSS Inc., Chicago, IL, USA). Means and standard deviations were computed for continuous data, and categorical data were assessed as frequencies. Each element’s overall ranking was calculated by averaging the forced rank data. Investigators were grouped into more- and less-experienced categories based on whether they had conducted CGI ratings in ≥20 or <20 previous studies, respectively. Similarly, for the analysis of time spent with patients, investigators were dichotomized into two groups: <20 versus ≥20 min. Comparisons of CGI ratings elements’ means between these groups were conducted with Mann-Whitney U test, and a Spearman’s correlation was used to examine relationships between the ranked elements for CGI-S ratings.

## 3. Results

Twenty-four investigators (20 physicians, 3 PhDs, and 1 missing response) completed the survey. The range of studies in which the investigators had performed CGI ratings spanned from 0 to 150 (median = 20). Ten investigators reported conducting ratings CGIs in <20 trials and 12 in ≥20 trials (two missing response). Thirteen (54.2%) investigators reported spending ≥20 min with patients at a mid-trial visit, and 11 (45.8%) spent <20 min. Reported interview length did not differ between investigators with greater or lesser CGI ratings experience (*p* = 0.868).

**Figure 1:**
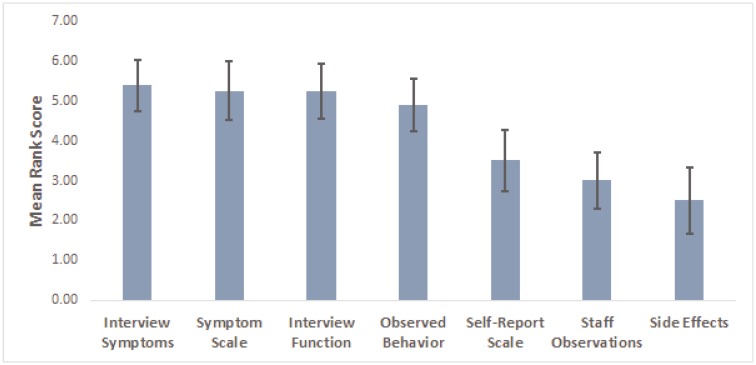
Mean ranking of elements contributing to CGI-S scoring. For purposes of illustration, the scoring has been reversed so that more important elements are represented by higher scores. Vertical bars represent 95% CI.

The mean rankings of elements used in determining CGI-S ratings are shown in Figure 1. The two most important elements, with near-equivalent mean ranked importance, were symptom severity based on the investigator’s interview and symptom score based on the objective clinician rating. Also highly ranked were functioning based on clinician interview and observable behaviors in the patient. Relatively little importance was placed on self-report symptom scores, staff observations, or side effects. The relative ranking of the elements contributing to CGI-S scoring did not differ by investigator experience (all *p* ≥ 0.159) or time spent with patients (all *p* ≥ 0.163). None of the investigators used the blank space on the questionnaire to indicate that they used any other type of information in formulating their CGI-S ratings.

Spearman’s correlations revealed moderate positive correlations between the rank assigned to objective scale scores and the rank assigned to subjective scale scores (rho = 0.425; *p* = 0.049), and between the rankings of observed behaviors and side effects (rho = 0.466; *p* = 0.029). There were no other clinically significant correlations between element rankings.

## 4. Discussion

In this examination of how investigators in clinical trials conduct CGI-S ratings, we found that the evaluation of clinical symptoms, whether by clinician-rating scale or by the investigator’s interview, was the most important element in determining CGI-S scores. The functional status of the patient emerged as a second important element in scoring. In contrast, low emphasis was given to self-report symptom scores, staff observations, or side effects. The relative importance of these CGI-S scoring elements was not significantly affected by the investigator’s experience or by the amount of time the investigator spent with the patient at a study visit. This consistency of CGI element emphasis between more- and less-experienced investigators in making CGI-S scores reflects the intuitive value of the CGI scales [15].

These results are consistent with prior research on investigators’ CGI ratings. Two prior studies found that investigators’ CGI scores were related more to rater-based scale scores than self-report scores [31,38]. In a study of patients with comorbid depressive and panic disorders, changes in symptom scores was the largest driver of CGI-S scores (38–40% of the variance) and CGI-I (26–40% of the variance) [31]. In a study of social anxiety disorder, CGI-S scores were driven primarily by self-reported social anxiety symptoms (28–55% of the variance), with clinician-rated assessments of symptom severity, depression, and impairment accounting for other significant proportions [33]. Similarly, in a pooled analysis of schizophrenia trials change in clinician-rated symptom severity was closely correlated with CGI-I scores [39].

Recently, Shear and colleagues demonstrated the value of a new form of psychotherapy, complicated grief treatment, using the CGI-I as the primary outcome in two large trials, in which the anchor points for scoring the CGI-S and CGI-I in patients with complicated grief were explicitly defined [25,26]. In addition to depressed mood, complicated grief is characterized by strong feelings of yearning for the deceased, avoidance of reminders of the deceased, and inability to accept the reality of the loved one’s death [25]. These symptoms were integrated into the CGI scoring anchors for complicated grief, which prevents the direct application of these anchors to studies of other psychiatric illnesses. However, the success and demonstrated inter-rater reliability of the structured CGI-I in these trials suggests that developing explicit CGI scoring anchors could improve the utility of the instrument in clinical treatment settings more broadly.

Advocates for measurement-based care acknowledge that treatment decisions cannot be based solely on symptom rating scale scores, and that measurement-based care is not a substitute for clinical judgment [40,41]. Patients consistently identify symptoms, functioning, and quality of life all as important treatment goals across psychiatric illnesses, suggesting that a transdiagnostic global measure capturing these factors may have clinical value. Thus, there is a need to develop a practical metric of clinical status that captures an understanding of the patient beyond that provided by symptom scales alone [42,43].

To meet this need, we created the Transdiagnostic CGI (T-CGI) (Table 2). To achieve the aims of cross-diagnostic application and improved reliability of ratings, we defined explicit scoring anchors for the CGI-S and CGI-I that did not depend on specific illness components. We created the anchors by comparing the results from the investigator’s survey with the published anchors for disease-specific CGI ratings. These comparisons found consistency between the factors emphasized by the investigators and the components included across the various disease-specific CGI anchors [19,20,21,22,23,24,25,26]. Of the disease-specific anchors, we considered those developed by Shear and colleagues for complicated grief to be the anchors most appropriately extended to a transdiagnostic version. Specifically, these anchors demonstrated the greatest integration between symptom level and functional status, and identified logical relationships between the CGI-S and CGI-I scores for individual patients [25,26]. Consequently, we modeled the T-CGI after the CGI modifications for complicated grief (Table 3). The T-CGI anchors intentionally describe functional status along with symptom burden for each level of scoring, and do not refer to specific symptoms other than suicidal thoughts, which should be considered for all severe psychiatric illnesses.

In addition to the flexibility to assess patients across a variety of diseases, an advantage of the T-CGI over symptom severity scales is that it allows the clinician to emphasize core symptoms over more peripheral symptoms. Most symptom rating scales do not differentially weight symptoms. For example, on the MADRS, a four-point reduction on the suicidality item counts the same as a four-point improvement in the sleep item, but a clinician would make an important distinction between the relative importance of those changes. The T-CGI measures may also prove useful for large pragmatic trials conducted in routine clinical settings, in which the time demands of symptom rating scales may be prohibitive [39,44].

The T-CGI may also find a role in clinical trials. Investigator meetings held in preparation for Phase II–IV regulatory trials typically focus on achieving inter-rater reliability for the primary outcome, typically a symptom-based scale, and neglect standardization for conducting CGI ratings. Given this absence of rater training, it is remarkable that the CGI measures have demonstrated good signal detection comparable to symptom rating scales in most trials [45,46]. Enhancing inter-rater reliability with training on detailed T-CGI scoring anchors would likely add to the utility of these assessments in trials, as has been demonstrated among clinical trialists applying the standard CGI [47]. The importance of the degree of functional impairment and changes in functioning when making T-CGI ratings should be emphasized as part of training on the instrument. Moreover, use of the T-CGI anchors we propose, both in clinical trials and in routine clinical environments, would substantially enhance the generalizability and interpretation of clinical trial findings, as the T-CGI-I scores would be based on the same standards in both settings. Given the simplicity of the T-CGI, training and evaluation could be completed with significantly less effort than currently goes in to the training and reliability assessment for the clinician-rated symptom scales.

There are some limitations to this study. The sample size of investigators was not large, and the rankings were not studied for replication in a separate sample of investigators. Differences between more- and less-experienced investigators may have emerged if the study had more power. In addition, the investigators were only asked about a hypothetical scenario with depressed patients. They were not asked about how they would rank items for other mental illnesses, though the existing literature suggests the same components emerge as most relevant across diseases. Whether the scale would perform adequately across patients and clinicians of varying cultural heritage is unknown. Finally, this study did not incorporate a prospective evaluation of the usefulness of our proposed T-CGI scoring anchors. Although the consistency in ranking of elements provides some reassurance about the evaluation of the CGI-S data, the study could not address the inter-rater reliability for actual CGI-S scores made for individual patients.

The next steps for the T-CGI will be to establish its validity and inter-rater reliability across a diversity of patient populations, both in terms of diagnostic profiles and sociodemographic characteristics. Convergent validity will be assessed by evaluating the correlation between T-CGI-S scores with scores from symptom rating scales for a patient’s primary psychiatric diagnosis. Similarly, to determine the T-CGI’s sensitivity to change, correlations between change on the symptom rating scale and T-CGI-I scores will be examined. Convergent validity will be evaluated using correlations between T-CGI scores and scales assessing functioning and quality of life. Because the T-CGI incorporates multiple aspects of the patient, we expect the T-CGI scores to correlate better with a combination score derived from symptom, functioning, and quality of life scales than with any scale assessing these individual components. Inter-rater reliability will be tested by having the same patient scored by different clinicians at the same visit.

## 5. Conclusions

The T-CGI aims to overcome the barriers to routine clinical implementation of the CGI arising from having separate scoring guides for each illness. Busy clinicians cannot be expected to learn and apply unique CGI anchors for each illness, but implementation of a unitary version with standard anchors across illnesses is an achievable goal. With the burgeoning of electronic medical records and the desire to mine clinical data, the T-CGI may offer value as an easily-captured metric of treatment efficacy [48].

## Figures and Tables

**Table 1:** Questionnaire for ranking components considered in making Clinical Global Impression-Severity (CGI-S) Scores.

Factor	Ranking (1–7)
Patient’s verbal report of symptom severity during interview	
Patient’s verbal report of their functional status	
Observable aspects of patient’s behavior	
Objective rating scale score (Hamilton, Montgomery-Asberg)	
Subjective rating scale scores (Beck, Inventory of Depressive Symptomatology)	
Degree of side-effects experienced by patient	
Comments from study coordinator or other study staff regarding their observations of the patient	
Other (Please write in):	

**Table 2:** Scoring anchors for the Transdiagnostic CGI-S and CGI-Improvement (CGI-I).

T-CGI-Severity		T-CGI-Improvement	
Severity Rating	Severity Level Description	Improvement Rating	Improvement Level Description
1. Normal	Symptoms are rarely present and occur only in contextually appropriate circumstances. The patient reports functioning at or very close to their full capacity.	1. Very much improved	Both the patient and the clinician agree that he/she has improved greatly from baseline, both in terms of symptoms and role functioning. The T-CGI-S score should be no more than mild (3), but in rare cases may be moderate (4) if the baseline severity was very high (7). If the T-CGI-S score is Normal (1), then the T-CGI-I score should be 1.
2. Borderline ill	Symptoms are few in number and only intermittently present, and usually no more than mild severity. There is little or no interference in role functioning.	2. Much improved	The patient has experienced clear and clinically meaningful reductions in symptoms, along with some improvement in role functioning, but distress or impairment from the illness persists. The T-CGI-S score should be no more than moderate (4), but in rare cases may be markedly ill (5) if the baseline severity was very high (7).
3. Mildly ill	Symptoms are clearly present and cause distress, but there is only minimal or no reduction in functioning.	3. Minimally improved	There is a detectable improvement in symptoms but little or no improvement in role functioning. The clinical significance of the changes is no more than minimal.
4. Moderately ill	Symptoms are present every day or nearly every day but may diminish at times. Substantial distress is present but bearable. Functioning in important roles is somewhat reduced, or maintained only through high levels of perceived effort. Suicidal thoughts may be present, but there is usually a desire to live.	4. No change	Symptoms and role functioning have not changed in any meaningful way since the baseline.
5. Markedly ill	Symptoms are highly distressing and the patient struggles greatly to function in important life roles. Active suicidal ideation may be present.	5. Minimally worse	There is a detectable worsening in symptoms but little or no change in role functioning. The clinical significance of the changes is no more than minimal.
6. Severely ill	Symptoms are nearly constant and highly distressing, and the patient is unable to function in important life roles. Active suicidal ideation may be present.	6. Much worse	Symptoms and role functioning are clearly worse from baseline. A change in treatment should be strongly considered.
7. Among the most extremely ill patients	Symptoms are continuously present at a very severe level. The person is unable to maintain basic functioning. Active suicidal thoughts are usually present. Hospitalization is usually required.	7. Very much worse	Symptoms and role functioning are dramatically worse than baseline. A change in treatment is definitely needed.

**Table 3:** Comparison of two complicated grief CGI scoring anchors ref. [26] with T-CGI scoring anchors.

**Complicated Grief CGI-S Moderately Ill (Score = 4)**	**T-CGI-S Moderately Ill (Score = 4)**
Symptoms of complicated grief are present and intrusive on most days at a level that is painful but bearable. There is some interference with activities and relationships, but functioning is not substantially impaired. There may be some avoidance of reminders of the loss. A sense of purpose or meaning is usually present, but there may be confusion about this. Suicidal thoughts may be present, but there is usually a desire to live.Distraction is possible temporarily, but symptoms are persistent and clinically significant.	Symptoms are present every day or nearly every day but may diminish at times. Substantial distress is present but bearable. Functioning in important roles is somewhat reduced, or maintained only through high levels of perceived effort. Suicidal thoughts may be present, but there is usually a desire to live.
**Complicated Grief CGI-I Much Improved (Score = 2)**	**T-CGI-I Much Improved (Score = 2)**
There is evidence that distress and impairment from CG are definitely improved compared with baseline, and this improvement is definitely clinically significant. The patient notices some difference in the role grief plays in her/his life. The CG-CGI-S score is usually no more than moderate (4). However, a patient can be much improved and grief symptoms may still be marked (5) if the baseline severity was very high (7).	The patient has experienced clear and clinically meaningful reductions in symptoms, along with some improvement in role functioning, but distress or impairment from the illness persists. The T-CGI-S score should be no more than moderate (4), but in rare cases may be markedly ill (5) if the baseline severity was very high (7).
